# Comparison of peak inspiratory flow rate via the Breezhaler®, Ellipta® and HandiHaler® dry powder inhalers in patients with moderate to very severe COPD: a randomized cross-over trial

**DOI:** 10.1186/s12890-018-0662-0

**Published:** 2018-06-14

**Authors:** Pablo Altman, Luis Wehbe, Juergen Dederichs, Tadhg Guerin, Brian Ament, Miguel Cardenas Moronta, Andrea Valeria Pino, Pankaj Goyal

**Affiliations:** 1 Novartis Pharmaceuticals Corporation, East Hanover, NJ USA; 2 Instituto Ave Pulmo, Fundación Enfisema, Mar del Plata, Argentina; 3 Novartis Pharma AG, Basel, Switzerland; 4 Novartis Ireland Limited, Dublin, Ireland; 5 Novartis Pharmaceuticals Corporation, San Carlos, California USA; 6 Novartis Argentina S.A, Buenos Aires, Argentina

**Keywords:** Peak inspiratory flow, Inspiratory effort, Dry powder inhalers, Pressure drop, Breezhaler®

## Abstract

**Background:**

The chronic and progressive nature of chronic obstructive pulmonary disease (COPD) requires self-administration of inhaled medication. Dry powder inhalers (DPIs) are increasingly being used for inhalation therapy in COPD. Important considerations when selecting DPIs include inhalation effort required and flow rates achieved by patients. Here, we present the comparison of the peak inspiratory flow rate (PIF) values achieved by COPD patients, with moderate to very severe airflow limitation, through the Breezhaler®, the Ellipta® and the HandiHaler® inhalers. The effects of disease severity, age and gender on PIF rate were also evaluated.

**Methods:**

This randomized, open-label, multicenter, cross-over, Phase IV study recruited patients with moderate to very severe airflow limitation (Global Initiative for Obstructive Lung Disease 2014 strategy), aged ≥40 years and having a smoking history of ≥10 pack years. No active drug or placebo was administered during the study. The inhalation profiles were recorded using inhalers fitted with a pressure tap and transducer at the wall of the mouthpiece. For each patient, the inhalation with the highest PIF value, out of three replicate inhalations per device, was selected for analysis. A paired t-test was performed to compare mean PIFs between each combination of devices.

**Results:**

In total, 97 COPD patients were enrolled and completed the study**.** The highest mean PIF value (L/min ± SE) was observed with the Breezhaler® (108 ± 23), followed by the Ellipta® (78 ± 15) and the HandiHaler® (49 ± 9) inhalers and the lowest mean pressure drop values were recorded with the Breezhaler® inhaler, followed by the Ellipta® inhaler and the HandiHaler® inhaler, in the overall patient population. A similar trend was consistently observed in patients across all subgroups of COPD severity, within all age groups and for both genders.

**Conclusions:**

Patients with COPD were able to inhale with the least inspiratory effort and generate the highest mean PIF value through the Breezhaler® inhaler when compared with the Ellipta® and the HandiHaler® inhalers. These results were similar irrespective of patients’ COPD severity, age or gender.

**Trial registration:**

The trial was registered with ClinicalTrials.gov NCT02596009 on 4 November 2015.

**Electronic supplementary material:**

The online version of this article (10.1186/s12890-018-0662-0) contains supplementary material, which is available to authorized users.

## Background

The progressive nature of chronic obstructive pulmonary disease (COPD), characterised by persistent airflow obstruction, necessitates regular self-administration of inhaled medications that are delivered directly to the desired site to relieve symptoms while minimizing systemic side effects [1]. Clinical trial and real-world study data provide conclusive evidence enabling physicians to make informed decisions about choice of medication [1]; however, little consideration is given to attributes of the inhaler and patients’ ability to use them [2], especially in elderly patients or those with severe disease [3]. The recently updated Global Initiative for Obstructive Lung Disease (GOLD) strategy document has taken a step towards reaching a consensus on considerations for ensuring effectiveness of the inhaled treatment [1]. However, there still seems to be a lack of agreement on considerations involved in choosing an appropriate inhaler.

A large variety of inhalers are currently available, each offering distinct advantages and disadvantages. Most metered-dose inhalers (MDIs) are not breath-actuated and use a pressurized propellant to deliver the drug, which means that less inspiratory effort is required. However, it is sometimes difficult for the patient to synchronize inhalation and actuation when using MDI devices [4, 5].

In contrast, breath-actuated dry powder inhalers (DPIs) [6] rely, amongst other factors, on the patient’s ability to produce sufficient airflow [7]. Each DPI has an intrinsic resistance that affects the inspiratory effort needed to effectively inhale the drug from the device [8]. The inspiratory maneuver of the patient creates a pressure differential within a DPI (reflective of the patient’s inspiratory effort), driving the airflow, which also depends on the intrinsic airflow resistance of the inhaler [9]. Inhalers with low airflow resistance allow air to flow through them more easily and are, therefore, more likely to allow the patients to inhale with a lower effort [10, 11] compared with higher resistance inhalers that require forceful inhalation [9]. An important consideration when choosing an inhaler, therefore, should be the ease associated with the inhalation effort required to take the medication [10]. Inhalation effort assumes even greater importance for COPD patients with muscular weakness, since ability to generate higher flow rates comfortably might be compromised, especially in patients with severe and very severe airflow limitation [9].

The Breezhaler® inhaler is a unit-dose, capsule-based DPI that has low internal (airflow) resistance [12, 13] is easy to use correctly, delivers a consistent dose of inhaled medication across different inhalation flow rates [14–17] and was suggested previously, through in-vitro studies, to require lower inhalation effort [10].

The objective of this study was to compare the peak inspiratory flow rate (PIF) values generated by patients with COPD through three different types of DPIs, the Breezhaler®, the Ellipta® and the HandiHaler®. The effects of disease severity, age and gender on PIF values generated through these inhalers were also evaluated.

## Methods

### Study design

**Fig. 1:**
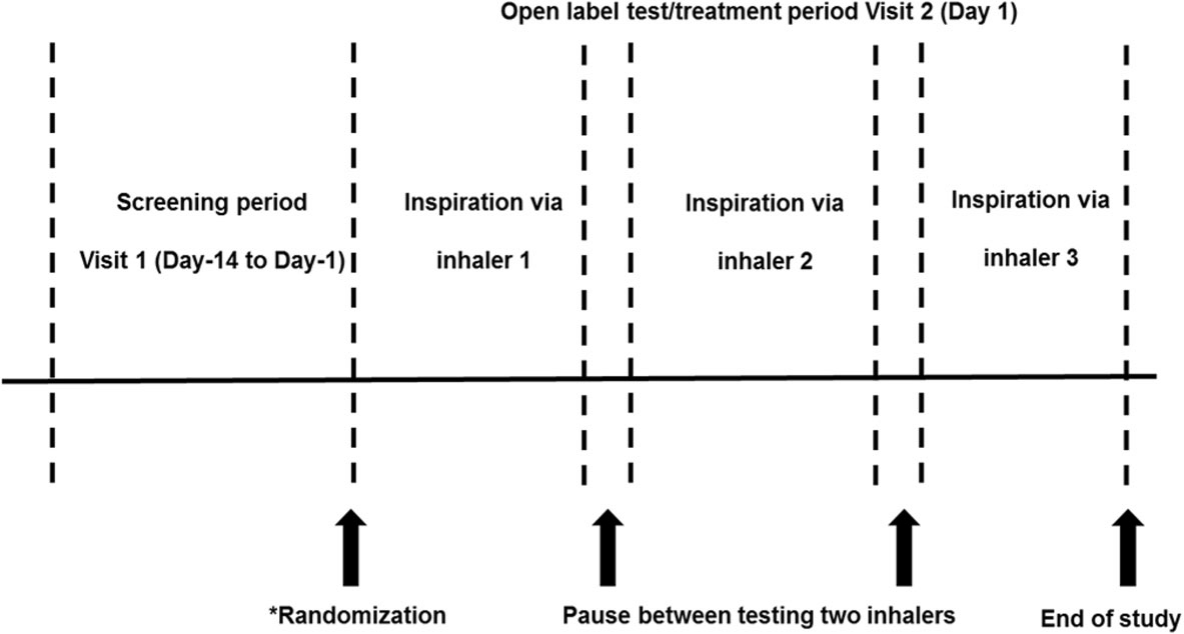
Study design. *Sequence of testing via inhaler 1, 2 and 3 for each patient will depend on randomization

This was a multicenter, open-label, randomized, cross-over, Phase IV study conducted across five sites in Argentina from 16 December 2015 to 29 April 2016 in COPD patients with moderate, severe, or very severe airflow limitation (GOLD 2014) [18]. There were two visits in the study: one for screening and the second for testing procedures (Fig. 1). The three inhalers tested in this study were the Breezhaler® (B), Ellipta® (E) and HandiHaler® (H) inhalers. No active drug or placebo was administered to patients during the study; the Breezhaler® and HandiHaler® inhalers with closed empty clear hydroxypropyl methylcellulose capsules were provided to each site and pierced just before inhalation measurements. The drug containing blister strips of Ellipta® were replaced by empty strips during device preparation by the investigator (Novartis). The inhalers were modified using a pressure tap and transducer fitted at the wall of the mouthpiece. The inhalational measurement method has been tested and validated by measurement of the airflow resistance of each device before and after the modification with the pressure tap. The airflow resistance was confirmed as unchanged due to the modification.

**Fig. 2:**
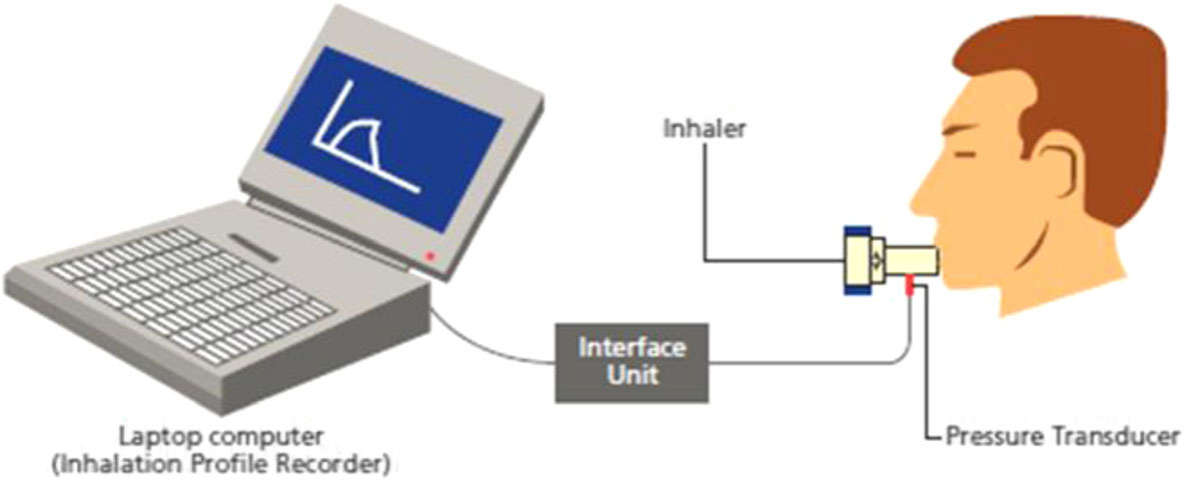
Setup to record inhalation profile of patients

Patients inhaled through the three DPIs following a randomized cross-over sequence (6 sequences used were: BEH, EHB, HBE, BHE, EBH and HEB). Before inhalation, patients were trained on use of each inhaler by the study personnel. A standard ‘Investigator’s Test Script’ was used across all sites to standardize the inhalation maneuver. Each patient was required to record three inhalation profiles via each inhalation device. Patients were allowed to rest between successive inhalations from the same device, and between inhalations from two different devices. The highest PIF value, of the three replicate inhalations per device, was used for the analyses. A custom-built and calibrated inhalation profile recorder (IPR; The Technology Partnership, UK) was used to measure and record patients’ inspiratory flow rates. Each inhaler was modified at the mouthpiece with a small stainless steel tube to connect to the IPR pressure transducer. The inhalers were characterized before the start of the study to ensure a specific pressure drop and the airflow through the device was comparable before and after attaching the instrumentation. Further, they were sanitized and packed for the study. Each device was given a unique identification number and label that were recorded in the case report form. During patient inhalation, the IPR measured the mouthpiece pressure drop, converted it into flow rate using inhaler resistance and plotted the results on the graphic–user interface (GUI) (Fig. 2). The PIF value was determined by the IPR software as the highest flow rate achieved by the patient and displayed on the GUI. The highest PIF value out of the three repeat runs per patient and per inhaler was used in the data analysis. Inhalation profiles were plotted using custom Matlab script according to the inhaler used and the subgroups: gender, age, and COPD severity, and manually reviewed.

### Ethics approval and consent to participate

The study protocol was reviewed by the institutional review boards and ethics committees for each center (list included in Additional file 1). The study was conducted according to the ethical principles of the Declaration of Helsinki. Written informed consent was obtained from each patient prior to performing any study related assessment.

### Participants

The study included men and women aged ≥40 years, diagnosed with moderate, severe, or very severe COPD, i.e. airflow limitation with post-bronchodilator forced expiratory volume in 1 s (FEV_1_) < 80% of predicted normal and FEV_1_/forced vital capacity < 0.70 at time of screening (GOLD 2014) [18]. Participants were current or ex-smokers with a smoking history of ≥10 pack years.

Patients were excluded if they experienced a COPD exacerbation that required treatment with antibiotics or oral corticosteroids or hospitalization within 6 weeks prior to screening, or had a respiratory tract infection within 4 weeks prior to screening, or had a history of asthma or onset of respiratory symptoms prior to the age of 40 years. Patients were also excluded if they were unable to use any of the three test inhalers due to cognitive impairment, neurological disorders, or any other condition affecting use of the DPIs.

### Study objectives

The primary objective of the study was to compare the PIF values generated by patients with moderate, severe, or very severe airflow obstruction through the Breezhaler®, Ellipta® and HandiHaler® DPIs. Exploratory objectives included assessing the effect of age, gender and disease severity on the PIF values generated through the three DPIs. Additional analyses included comparison of the pressure drop at PIF (as a measure of inspiratory effort) across all devices as a function of COPD severity, age (40–64, 65–74 and ≥ 75) and gender.

### Assessments

Raw data included the pressure drop values, and flow rate was calculated using the equation:

√ΔP = Q * R where ΔP (kPa) is the pressure drop observed in the inhaler, Q (L/min) is the inhalation flow and R (cmH_2_O^0.5^[L/min]^− 1^) is the airflow resistance [9]. To convert pressure drop to flow rate, previously published ‘measured internal resistance’ values for Breezhaler®, Ellipta® and HandiHaler® inhalers (i.e. 0.060, 0.090 and 0.163 cmH_2_O^0.5^[L/min]^− 1^ [19, 20]) respectively, were used. Pressure drops at PIF were compared between the three inhalers as a function of COPD severity, age and gender. Safety was evaluated based on the incidence rate of adverse events (AEs), vital signs, and physical examination.

### Statistical analysis

The full analysis set consisted of all 97 patients who conducted an inhalation maneuver through at least one inhaler. Profiles containing constant pressure values, termed ‘flat lines’, evaluated as incorrect profiles, were not considered in the final analysis. *P*-values were generated using a paired t-test with *P* < 0.01 indicating a significant difference which controls the familywise type 1 error rate at 3% under multiple testing for the primary PIF analysis on the FAS. *P*-values for subgroup analysis are exploratory in nature.

### Sample size

A six-sequence Williams design was used to calculate the sample size. This design and an assumed standard deviation (SD) of 17.7 L/min (calculated according to previous findings) and a sample size of 96 patients (16 in each of the six possible sequences, i.e. 16 × 6 = 96) had > 90% power to detect a difference in PIF values between a pair of inhalers of 10 L/min (2-sided alpha 0.05).

## Results

### Participants

A total of 97 patients with a mean ± SD age of 69.0 ± 8.2 years enrolled and completed the study. Almost all patients were Caucasians and the majority of the population was male. The majority of patients had either moderate or severe COPD with mean ± SD time since diagnosis of 8.2 ± 4.2 years (Table 1). Overall, 15.5% of patients had experienced a COPD exacerbation in the previous year.

**Table 1:** Baseline demographics and clinical characteristics (Full analysis set)

Characteristic	Value (*N* = 97)
Age, years	69.0 ± 8.2
Male, *n* (%)	72 (74.2)
Race, *n* (%)	
Caucasian	96 (99.0)
Asian	1 (1.0)
BMI (kg/m^2^)	27.0 ± 5.3
Current smoker, n (%)	14 (14.4)
Severity of COPD, n (%)	
Moderate	49 (50.5)
Severe	38 (39.2)
Very severe	10 (10.3)
Number of COPD exacerbations in the previous year	0.2 ± 0.51
Post-bronchodilator FEV_1_, % predicted	50.7 ± 15.5
Post-bronchodilator (%) FEV_1_/FVC	48.3 ± 11.3

Data are presented as mean ± standard deviation, unless otherwise specified. COPD severity is based on GOLD 2014 criteria

*BMI* body mass index, *FEV*_1_ forced expiratory volume in 1 s, *FVC* forced vital capacity, *GOLD* global initiative for chronic obstructive lung disease

### PIF values and pressure drop

In the overall population, patients produced the highest mean PIF values with the Breezhaler® inhaler followed by the Ellipta® and the HandiHaler® inhalers. The lowest mean pressure drop values were recorded with the Breezhaler® inhaler, again followed by the Ellipta® inhaler and the HandiHaler® inhaler (Table 2).

**Table 2:** Comparison of mean PIF and pressure drop values in overall population

Variable	Breezhaler®	Ellipta®	HandiHaler®
*n*	97	91	97
R (cmH_2_O^0.5^[L/min]^−1^)^a^	0.060	0.090	0.163
PIF (L/min)	108 ± 23	78 ± 15	49 ± 9
Range (Min-Max)	54–156	45–109	22–70
Pressure drop at PIF (cmH_2_O)	44 ± 18	51 ± 19	67 ± 23
ΔPIF vs Breezhaler® (L/min); 95% CI	–	30; 27 to 32	59; 56 to 62
*P*-value		< 0.0001	< 0.0001

Data are presented as mean ± standard deviation unless stated otherwise. *P*-values generated from a paired t-test on comparison of PIF values

Poor quality (flat-line) inhalational profiles with erroneous PIF values were not considered in the analysis

*CI* confidence interval, *n* number of patients, *ΔPIF* difference in mean PIF values, *PIF* peak inspiratory flow rate, R intrinsic airflow resistance of the inhaler

^a^Data published previously [19, 20]

### Subgroup analysis by severity of COPD

The results observed across all COPD severities were in line with the overall population; i.e. patients of all subgroups produced highest mean PIF values with the Breezhaler® compared with the Ellipta® and the HandiHaler® inhalers. The lowest mean pressure drop values were observed for the Breezhaler® followed by the Ellipta® and the HandiHaler® inhalers (Table 3). For each inhaler, the mean PIF values were similar for patients with moderate and severe COPD, but lower for patients with very severe COPD (Table 3).

**Table 3:** Comparison of mean PIF and pressure drop values based on severity of COPD

Severity	Variable	Breezhaler®	Ellipta®	HandiHaler®
	R (cmH_2_O^0.5^[L/min]^− 1^)^a^	0.060	0.090	0.163
Moderate	*n*	49	44	49
	PIF (L/min)	109 ± 26	78 ± 15	50 ± 10
	Range (Min–Max)	54–152	45–102	22–68
	Pressure drop at PIF (cmH_2_O)	45 ± 20	52 ± 19	68 ± 25
	ΔPIF vs Breezhaler® (L/min); 95% CI	–	31; 26 to 34	59; 54 to 64
	*P*-value		< 0.0001	< 0.0001
Severe	*n*	38	37	38
	PIF (L/min)	110 ± 22	79 ± 15	49 ± 8
	Range (Min–Max)	71–156	48–109	31–70
	Pressure drop at PIF (cmH_2_O)	45 ± 17	53 ± 19	67 ± 22
	ΔPIF vs Breezhaler® (L/min); 95% CI	–	31; 26 to 33	61; 55 to 65
	*P*-value	–	< 0.0001	< 0.0001
Very severe	*n*	10	10	10
	PIF (L/min)	99 ± 14	71 ± 13	46 ± 7
	Range (Min–Max)	77–128	52–102	33–59
	Pressure drop at PIF (cmH_2_O)	36 ± 10	42 ± 17	58 ± 17
	ΔPIF vs Breezhaler® (L/min); 95% CI	–	28; 24 to 32	53; 46 to 60
	*P*-value	–	< 0.0001	< 0.0001

Data are presented as mean ± standard deviation unless stated otherwise. *P*-values for subgroup analysis are exploratory in nature

Poor quality (flat-line) inhalational profiles with erroneous PIF values were not considered in the analysis

*CI* confidence interval, *n* number of patients, *ΔPIF* difference in mean PIF values, *PIF* peak inspiratory flow rate, *R* intrinsic airflow resistance of the inhaler

^a^Data published previously [19, 20]

### Subgroup analysis by age

Results observed across all age groups were also consistent with the overall results. Irrespective of age group, patients produced highest mean PIF values with the Breezhaler® versus the Ellipta® and the HandiHaler® inhalers, and the lowest mean pressure drop values were recorded for the Breezhaler® versus the Ellipta® and the HandiHaler® inhalers (Table 4). Across all inhalers, patients aged 40–64 years generated the highest mean PIF values, while patients aged ≥75 years generated mean PIF values comparable with those aged 65–74 years (Table 4).

**Table 4:** Comparison of mean PIF and pressure drop values based on age

Age (years)	Variable	Breezhaler®	Ellipta®	HandiHaler®
	R (cmH_2_O^0.5^[L/min]^− 1^)^a^	0.060	0.090	0.163
40–64	*n*	27	27	27
	PIF (L/min)	123 ± 20	88 ± 13	53 ± 9
	Range (Min-Max)	77–156	57–109	32–70
	Pressure drop at PIF (cmH_2_O)	56 ± 17	64 ± 18	76 ± 23
	ΔPIF vs Breezhaler® (L/min); 95% CI	–	35; 30 to 40	70; 65 to 76
	*P*-value	–	< 0.0001	< 0.0001
65–74	*n*	46	42	46
	PIF (L/min)	101 ± 22	73 ± 13	47 ± 9
	Range (Min-Max)	54–152	48–99	22–68
	Pressure drop at PIF (cmH_2_O)	38 ± 16	45 ± 16	61 ± 23
	ΔPIF vs Breezhaler® (L/min); 95% CI	–	28; 23 to 30	54; 49 to 58
	*P*-value	–	< 0.0001	< 0.0001
≥75	*n*	24	22	24
	PIF (L/min)	105 ± 21	75 ± 16	50 ± 9
	Range (Min-Max)	65–151	45–102	29–66
	Pressure drop at PIF (cmH_2_O)	41 ± 17	48 ± 18	67 ± 22
	ΔPIF vs Breezhaler® (L/min); 95% CI	–	30; 24 to 35	55; 49 to 62
	*P*-value	–	< 0.0001	< 0.0001

Data are presented as mean ± standard deviation unless stated otherwise. *P*-values for subgroup analysis are exploratory in nature

*CI* confidence interval, *n* number of patients, *ΔPIF* difference in mean PIF values, *PIF* peak inspiratory flow rate, *R* intrinsic airflow resistance of the inhaler

Poor quality (flat-line) inhalational profiles with erroneous PIF values were not considered in the analysis

^a^Data published previously [19, 20]

### Subgroup analysis by gender

Similar to the overall results, both male and female patient populations produced the highest mean PIF values with the Breezhaler® compared with the Ellipta® and the HandiHaler® inhalers. The lowest mean pressure drop values were observed for the Breezhaler® versus the Ellipta® and the HandiHaler® inhalers, irrespective of gender (Table 5). Irrespective of the inhaler used, the PIF values observed for females were consistently lower than those observed for males (Table 5).

**Table 5:** Comparison of mean PIF and pressure drop values based on gender

Gender		Breezhaler®	Ellipta®	HandiHaler®
	R (cmH_2_O^0.5^[L/min]^−1^)^a^	0.060	0.090	0.163
Male	*n*	72	68	72
	PIF (L/min)	111 ± 24	81 ± 16	51 ± 9
	Range (Min-Max)	54–156	45–109	22–70
	Pressure drop at PIF (cmH_2_O)	47 ± 19	54 ± 20	71 ± 23
	ΔPIF vs Breezhaler® (L/min); 95% CI	–	30; 28 to 34	60; 57 to 65
	*P*-value	–	< 0.0001	< 0.0001
Female	*n*	25	23	25
	PIF (L/min)	98 ± 15	71 ± 11	45 ± 8
	Range (Min-Max)	71–121	49–89	32–57
	Pressure drop at PIF (cmH_2_O)	36 ± 11	41 ± 12	56 ± 20
	ΔPIF vs Breezhaler® (L/min); 95% CI	–	27; 23 to 31	53; 49 to 57
	*P*-value	–	< 0.0001	< 0.0001

Data are presented as mean ± standard deviation unless stated otherwise. *P-*values for subgroup analysis are exploratory in nature

*CI* confidence interval, *n* number of patients, *ΔPIF* difference in mean PIF values, *PIF* peak inspiratory flow rate, *R* intrinsic airflow resistance of the inhaler

Poor quality (flat-line) inhalational profiles with erroneous PIF values were not considered in the analysis

^a^Data published previously [19, 20]

### Safety

No AEs or serious AEs were reported in this study. No clinically significant observations were observed with regards to changes in vital signs.

## Discussion

The clinical relevance of inhaled therapy relies on its ability to deliver drug directly to the intended site and avoiding systemic side effects [1]. While identifying the most appropriate treatment, health-care professionals are often more focused on pharmacological properties of a drug and tend to overlook, that inhaler characteristics may have an impact on the overall treatment benefit [21]. Patients’ inhalation flow pattern can significantly influence the performance of an inhaler, thus impact effectiveness of the inhalation therapy [22]. However, there is limited data available comparing the inspiratory flow rates of the marketed products [17]. Our study provides direct comparison of inspiratory flows achieved by COPD patients between the three widely used dry powder inhalers i.e. the Breezhaler®, the Ellipta® and the HandiHaler® with varying internal resistance.

It is important to assess patients’ characteristics when prescribing the medication. Patient characteristics such as age, gender and disease severity can affect the performance of the inhalers; studies have reported that increasing age [23] and COPD severity [24], may reduce patients’ inhalation capability. Mahler et al. observed that suboptimal PIF values were predominantly exhibited by female patients when using DPI [25].

In this study, COPD patients produced the highest mean PIF rate when inhaling through the Breezhaler® inhaler compared with the Ellipta® and the HandiHaler® inhalers. Correspondingly, the lowest mean pressure drop was recorded when patients were using the Breezhaler® inhaler versus the Ellipta® or the HandiHaler® inhalers. This confirmed the prior knowledge that for a set inspiratory effort, the inhalation flows when using a low resistance DPI are expected be greater than when using a DPI with a higher resistance [26].

The mechanism of breath actuation in DPIs requires patients to generate airflow against the intrinsic airflow resistance of the inhaler. The reliance on patient-generated flow rate for effective inhalation can be minimized when using an inhaler with low intrinsic airflow resistance [11], so that patients need to exert lower inspiratory effort to generate sufficient and sustained airflow [8]. In this study, patients required least inspiratory effort when inhaling through the Breezhaler® compared with the Ellipta® and the HandiHaler® DPIs, suggesting that patients can inhale more comfortably though low resistance Breezhaler® inhaler. The results were similar irrespective of patients’ age, gender or COPD severity.

Additionally, it has been previously shown that at pressure drop of up to 4 kPa (equivalent to 41 cmH_2_O), patients are able to inhale comfortably through a DPI [27]. In this study, a pressure drop of 41 cmH_2_O and 36 cmH_2_O at PIF was achieved by patients of older age (≥75 years) and patients with very severe COPD, respectively, when using the Breezhaler® inhaler compared with either the Ellipta® or the HandiHaler® inhalers. The inspiratory effort determined for the Breezhaler® inhaler was less than 4 kPa at any estimated flow rate [28], indicating that patients inhaled most comfortably when using this device.

The inspiratory efforts required by patients’ becomes an important consideration in treatment decisions, when a minimum inhalation flow through a specific DPI is critical for efficient de-aggregation of drug particles [26] which is not the case with the low resistance DPIs like Breezhaler® inhaler. Previously published studies for glycopyrronium and indacaterol via Breezhaler® demonstrated there was consistent dose delivery performance from Breezhaler® using airflow rates between 50 and 100 L/min. In our present study also in overall population and in the sub-groups, by COPD severity or age or gender, all patients were able to generate PIF rates of at least 54 L/min through Breezhaler, suggesting consistent dose delivery performance from Breezhaler® irrespective of patients’ age, gender or COPD severity.

Some COPD patients may have difficulty with the effort required to generate sufficient inspiratory flow through a high-resistance inhaler because of loss of muscle mass and/or COPD severity [29, 30]. Therefore, when prescribing an inhalation treatment, it is important to establish whether patients can inhale comfortably through the inhaler device to be used to deliver that treatment. The ease with which patients are able to inhale through an inhaler is an important aspect of COPD management. Our results show that patients, irrespective of their COPD severity, age and gender, were able to inhale with least inspiratory effort (pressure drop) and generate highest PIF values when inhaling through the Breezhaler® inhaler compared with either the Ellipta® or the HandiHaler® inhalers. Although, we observed a slightly aberrant pattern with higher PIF values in patients aged ≥75 years (105 L/min) than that in patients aged 65–74 years (101 L/min), this could be due to comparatively less number of patients analyzed in ≥75 years age group (*n* = 24 versus *n* = 46 in 65–74 years) in the study.

The strengths of this study include minimization of inter-subject variability as measures like PIF or pressure drop are objective physical and mathematical measures, which may not be affected by prior use or experience of the patients unlike handling, preference or other measures. Furthermore random allocation and cross over design further minimizes any potential sequence bias. This study had certain limitations: the pharmacological effect of drug inhalation was not studied because this study design required multiple inhalations through the inhalers over short period of time and the PIF values may not reflect a non-research setting such as at home or during an exacerbation.

Information on the comparative inhalational flow rates and inhalation profiles from the studies DPIs and across the various studies COPD patients sub-groups would allow physicians to make informed decisions in selecting the right inhaler for the patient. Further studies would be useful to establish the generalizability of these results.

## Conclusion

The results showed that mean PIF values increased and mean pressure drop values at the PIF decreased with decreasing airflow resistance of the inhalers. Patients with COPD were able to inhale with the least inspiratory effort and generate the highest mean PIF value through the Breezhaler® inhaler when compared with the Ellipta® inhaler and the HandiHaler® inhaler. The results were similar irrespective of patients’ COPD severity, age or gender.

## Additional file

Additional file 1: List of Independent Ethics Committees. (DOCX 14 kb)

## Abbreviations

AE: Adverse event
BMI: Body mass index
DPI: Dry-powder inhaler
FEV_1_: Forced expiratory volume in 1 s
GOLD: Global initiative for obstructive lung disease
GUI: Graphic user interface
IPR: Inhalation profile recorder
MDI: Metered-dose inhaler
PIF: Peak inspiratory flow rate
SAE: Serious adverse event
SD: Standard deviation
