# Supplementary material for: Comparison of peak inspiratory flow rate via the Breezhaler®, Ellipta® and HandiHaler® dry powder inhalers in patients with moderate to very severe COPD: a randomized cross-over trial
Source: BMC Pulm Med. 2018 Jun 14;18:100. doi: 10.1186/s12890-018-0662-0 (PMC6001060; doi:10.1186/s12890-018-0662-0)
Supplement: Supplementary file 1 — List of Independent Ethics Committees. (DOCX 14 kb) [file 12890_2018_662_MOESM1_ESM.docx]

**List of Independent Ethics Committees or Institutional Review Boards**

| **Center No.** | **Ethics Committee or**  **Institutional Review Board** | **Department / Organization** | **Address**  **Country** |
| --- | --- | --- | --- |
| **0001** | Comité de Revisión Interna | Naciones Unidas 346 | Córdoba Córdoba X5016KEH  Argentina |
| **0002** | Comité Independiente de  Ëtica | Uriburu 774 1st floor | CABA Buenos Aires C1027AAP  Argentina |
| **0003** | Comité Independiente de  Ëtica | Uriburu 774 1st floor | CABA Buenos Aires C1027AAP  Argentina |
| **0004** | Comité Independiente de  Ëtica | Uriburu 774 1st floor | CABA Buenos Aires C1027AAP  Argentina |
| **0005** | Comité Independiente de  Ëtica | Uriburu 774 1st floor | CABA Buenos Aires C1027AAP  Argentina |
| **0006** | Comité Independiente de  Ëtica | Uriburu 774 1st floor | CABA Buenos Aires C1027AAP  Argentina |
| **0008** | Comité Independiente de  Ëtica | Uriburu 774 1st floor | CABA Buenos Aires C1027AAP  Argentina |
| **0009** | Comité de Ética en  Investigación | Av. Colón 3364 | Mar del Plata Buenos Aires  B7600FZN  Argentina |
| **0010** | Comité Independiente de  Ëtica | Uriburu 774 1st floor | CABA Buenos Aires C1027AAP  Argentina |
